# Evaluating teacher multilingualism across contexts and multiple languages: validation and insights^☆^

**DOI:** 10.1016/j.heliyon.2020.e04471

**Published:** 2020-08-07

**Authors:** Raees Calafato

**Affiliations:** Department of Foreign Languages, University of Bergen, HF-bygget, Sydnesplassen 7, 5020 Bergen, Norway

**Keywords:** Education, Multilingualism, Teacher education, Teacher practices, Evaluation, Teacher beliefs, Language teaching, Second language acquisition

## Abstract

In response to the need for quantitative instruments that can provide insights into language teacher multilingualism on a large scale, this article discusses the development of the MULTITEACH questionnaire via a five-stage process that consisted of a critical review of research on teacher multilingualism, seminar and practitioner consultations, a pilot study, reliability tests, and principle component analysis (PCA), followed by a larger study involving 460 multilingual language teachers and factor analysis to confirm the PCA. The questionnaire thus validated provides a comprehensive quantitative measure of assessing language teacher multilingualism across multiple foreign languages and in diverse contexts. A discussion of the factors influencing teacher multilingualism has also been included based on the findings from the larger study of 460 multilingual teachers.

## 1 Introduction

Research on multilingualism as a learning and teaching resource has been growing steadily in the last few years in tandem with initiatives taken by regional blocs and individual countries to promote the learning of multiple languages, especially in secondary education (Byram, 2018). Such initiatives reflect the increasingly super-diverse character of many cities and countries around the world (Spotti and Kroon, 2017), where hundreds of nationalities live together and interactions occur in diverse languages. For example, schools in the United Arab Emirates often promote the learning of more than one foreign language (FL) among their students, who end up learning three FLs simultaneously (Calafato and Tang, 2019). In Russia, a recent initiative by the Ministry of Education (MoE) gives students the opportunity to acquire two FLs by the time they graduate from school (MoE Russia, 2018), similar to the system of FL education in Norway (Haukås, 2016). And while many multilingual initiatives have traditionally focused on the learning of English as a foreign (EFL) or second language (ESL), this, too, appears to be changing. One example of this change is Saudi Arabia's recent announcement that Mandarin Chinese will be taught at all stages of secondary and tertiary education (Al Arabiya, 2019). Taken together, these developments can be considered portents of how language education is set to change due to the evolving nature of geopolitics, economic ties, and the strategic interests of nations and blocs.

Given the growing importance of developing multilingual citizens in an increasingly multipolar world, countries implementing multilingual educational initiatives have to contend with not only designing effective curricula that will lead to successful learning outcomes among learners but they also need to train and evaluate language teachers in order to ensure that these develop and continue to nurture the skills and beliefs needed to promote their students' desire to be multilingual. Teachers who are not multilingual themselves and who do not desire to be multilingual may not be able to inspire their students to develop a multilingual identity. Their beliefs might be influenced by teacher education programs, which sometimes promote a monolingual approach to teaching languages and pay little attention to teachers' (and students') multilingualism (Ellis, 2016; Otwinowska, 2017). Nationalism could be another reason for why teachers adopt a monolingual approach to teaching languages. For instance, they might perceive minority and immigrant languages as having less value (see Bourdieu, 1990) than the national language (Pulinx et al., 2017). The continued use of a monolingual approach to language teaching in many countries has led some writers to call for requiring language teachers to learn a new language as part of teacher education programs because they believe that teachers will benefit from the experiential knowledge thus acquired (Ellis, 2016).

Such an addition to teacher education programs could lead to some teachers adopting a multilingual pedagogy (see Haukås, 2016); however, it is important to first evaluate what teachers believe about multilingualism as a resource for the teaching and learning of languages and what factors may influence them to draw on their and their students' knowledge of other languages during lessons. Such an evaluation would help educational institutions make targeted, informed changes to teacher education programs to help teachers meet the challenges of educating an increasingly multilingual and super-diverse student population based on empirical, first-hand research. In this respect, a growing number of studies have explored teacher multilingualism, although there is a lack of systematization when it comes to methodology and a preponderance of qualitative studies (see Calafato, 2019). The lack of systematization makes it difficult to compare teacher multilingualism across diverse contexts and languages. One way to better systematize research would be through the development of evaluative quantitative instruments that have been validated in terms of content, criterion, and construct (see Newton and Shaw, 2014), with input from teachers and educationalists. In addition, these instruments would need to provide a sufficient level of reliability with respect to the teaching of diverse languages in different countries (see Calafato, 2019) and be comprehensive enough to explore the various facets of teacher multilingualism while also allowing for the convenient surveying of large numbers of teachers.

Currently, there appear to be few such quantitative instruments and fewer still have been used in multiple studies. This study discusses the processes, materials, and validating techniques used to create and test an online questionnaire (MULTITEACH) for assessing language teacher multilingualism, specifically, their beliefs about the learning and teaching of multiple languages and their use of multilingual practices in the classroom, operationalized through the ways in which teachers perceive, and benefit from, their multilingual affordances (Kordt, 2018) and abilities (Jessner, 2018).

## 2 Background to the instrument

### 2.1 The multilingual turn in teaching languages: Theoretical considerations

State promotion of multilingualism, which is the use of more than one language by individuals, societies, institutions, and groups in daily life (European Commission, 2007; see also Clyne, 2017; Kemp, 2009), is partly predicated on the belief that it improves one's cognitive abilities (Hirosh and Degani, 2018), mathematical learning (Dahm and De Angelis, 2018), creativity (Fürst and Grin, 2018), and cultural knowledge (Ellis, 2016). A more multilingual society can also lead to increased understanding, better relations, and openness between countries on both the individual and state levels. With respect to the teaching and learning of languages, multilingualism and its potential benefits can be approached using a variety of theoretical frameworks. Jessner (2006, 2008), applying Dynamic Systems Theory to the learning of languages by multilinguals, writes that such individuals develop multilingual systems, composed of language-specific and non-language-specific skills and abilities related to language learning, management, and maintenance, which are not available to monolinguals. She posits that these systems positively influence multilinguals' creative thinking, communicative sensitivity, flexibility, translation skills, and interactional and pragmatic competence.

In much the same way, multilingual language teachers (MLTs) can be said to possess several skills and abilities that they can use to support and enhance their teaching practices in ways that might not be possible for teachers that have not developed these multilingual systems (Calafato, 2019). Another framework that one can use to conceptualize MLTs is the theory of multi-competence (Cook, 2008, 2012), which covers an individual or community's knowledge of more than one language, taking into account the sum total, as well as interrelationships, of all the languages and how these can affect not only language use but also the mind in general. Their multi-competence could furnish MLTs with greater cognitive flexibility and metalinguistic awareness, which they would use to engage their students in a deeper exploration of how languages function (see ). Such an exploration, when viewed through an ecological analysis approach Kramsch and Whiteside (2008), which posits that multilinguals display behavior that exceeds mere communicative competence in interactions, may benefit students not only in terms of language learning but also in other life domains.

According to Kramsch and Whiteside (2008, p. 664), the multilingual individual has “a particularly acute ability to play with various linguistic codes and with the various spatial and temporal resonances of these codes”. They term this ‘symbolic competence’, defining it as “the ability not only to approximate or appropriate for oneself someone else's language, but to shape the very context in which the language is learned and used” (p. 664). Symbolic competence ties in well with the adaptation by Singleton and Aronin (2007) of the theory of affordances Gibson (1977) to describe multilingualism. With respect to languages, affordances represent what languages offer, in tangible and intangible ways, to the individual in terms of cognitive and evaluative abilities, emotions, and knowledge (Aronin, 2014). The use of these affordances entails a heightened awareness on the part of multilingual people, including teachers, of the many ways in which they can interact with their environment. The greater the number of languages that an individual knows, the greater the number of affordances he or she enjoys, although empirical research in support of this is still difficult to find. In short, all these frameworks suggest that MLTs have a *potentially* rich and complex toolset that they can use to enhance their teaching in diverse ways.

### 2.2 Empirical studies on multilingual teachers

While theoretical frameworks are useful in providing a better understanding of the *potential* that MLTs possess, it is important to also ascertain whether these theories are supported by evidence from research on actual MLTs. In this regard, studies tend to report a mixed picture and generally fall into three main categories with respect to MLTs: (1) teacher beliefs and professional identity, (2) teaching practices, and (3) the beliefs of other stakeholders towards teacher multilingualism (for a review of some such studies, see Calafato, 2019).

#### 2.2.1 Teacher beliefs, identity, and practice

In terms of teacher beliefs and identity, studies indicate various levels of acceptance by language teachers of their multilingualism. At one extreme, teachers reject their multilingualism in favor of a monolingual ideal (e.g. Zheng, 2017), where they prefer to act as if they only knew and spoke the target language. In doing so, they risk imposing an immersive experience on their students that discourages them from using their prior language experiences and knowledge as a learning resource. At the other end, not only do they embrace their multilingualism but they also show pride in it when interacting with their students and other stakeholders (e.g. Ng, 2018). There is some indication that the environment and teachers' experiences as language learners can strongly influence how they end up expressing their multilingualism. For instance, teachers in Asia, the United Kingdom, and the Americas can find it more challenging to express their multilingualism due to the widespread presence of societal beliefs that link legitimacy as a speaker of a certain language to ethnicity or nationality (Calafato, 2019). In other words, the most proficient and authentic speakers of a language in these regions are often seen as those that were born into it (nativeness vs. later acquisition). In contrast, the situation in Europe (excluding the United Kingdom) is more accepting of multilingualism (European Union, 2011), although issues of teacher legitimacy do arise (see Kramsch and Zhang, 2018). Studies also show that while teachers express positive beliefs about multilingualism, irrespective of context, they do not always engage in multilingual practices with their students (e.g. Haukås, 2016) or understand how languages interact in the multilingual mind (De Angelis, 2011; Otwinowska, 2014).

The implication is that teachers' beliefs about multilingualism can exist separately from their practices since positive beliefs do not necessarily translate into concrete actions. This disconnect between their beliefs and practices may be due to teacher education programs in many countries lacking an explicit component that introduces teachers to the practical implementation of a multilingual pedagogy (Otwinowska, 2017). The result is that teachers may not have the tools to convert their beliefs into practice (Schedel and Bonvin, 2017). For example, in the absence of appropriate training, teachers' decision to engage in multilingual practices has been shown to largely depend on their personal experiences and reaction to native-speakerism (see Calafato, 2019; Jiang et al., 2014). Oftentimes, teachers embark on a period of self-reflection and independent study before they acquire the tools and confidence to implement a multilingual pedagogy (e.g. Ng, 2018). As for the specific multilingual practices that MLTs have been found to implement, studies show that they draw on cross-linguistic comparisons to enhance the teaching of vocabulary and grammar, and use translanguaging techniques and collaborative learning (Calafato, 2019; Figueiredo, 2011; Matsumoto, 2018; Pahissa and Tragant, 2009). The students and other stakeholders (the school administration, parents, etc.) in many such studies corroborate the positive effects of these practices on the language learning experience (see Figueiredo, 2011), especially on the students' ability to use language at a more advanced level and on their writing skills, both of which require greater cognitive input (Mahboob and Lin, 2016).

#### 2.2.2 Instruments for collecting data on multilingual teachers

Studies on multilingual language teachers have mostly explored their identity, beliefs, and practices via qualitative instruments, usually in the form of interviews, discussion groups, and observations (see Calafato, 2019). Fewer studies have employed quantitative measures that have sampled a large pool of MLTs. While qualitative studies can offer valuable insights into the ways in which MLTs perceive and make use of their affordances, the small number of participants in such studies makes it difficult to generalize the findings to the wider community of such teachers in a given context (Calafato, 2019). Qualitative research can also often lack organization and replicability, with most studies being one-offs. The result is that it is not clear if similar findings could be obtained in different contexts using the same methods. Finally, few studies, regardless of the research method used, have studied the multilingualism of teachers of languages other than English (LOTEs). This represents a significant gap in our knowledge of teacher multilingualism since English can be taught differently than LOTEs. For example, students start to learn English soon after they enter primary school in several countries and are often taught English more communicatively than they are LOTEs, which they start learning in later grades (see Haukås, 2016). The order in which students learn FLs might influence the practices of their English and LOTE teachers regarding the implementation of a multilingual pedagogy in the classroom and whether the teachers consider it beneficial to draw on their students' knowledge of other languages. There is also a dearth of instruments that have been designed to explore the multilingualism of teachers who teach multiple languages. For instance, it is possible that these teachers possess different levels of proficiency per FL taught, based on their language learning experiences at school, university, or in other contexts, which may then affect how they go on to teach the FLs in their repertoire (see Aslan, 2015).

## 3 Methods

In seeking to validate the MULTITEACH questionnaire (for the final version, see Appendix A), the main goal was to assess if the various questionnaire items measured what they were meant to. It was also necessary to use different types of validity in order to ensure that the inferences made from the questionnaire data accurately reflected the beliefs and teaching practices of the multilingual participants being studied. As a result, the validation process consisted of construct validation (how well the instrument measures the constructs it seeks to explore; this type of validation can be done by conducting factor analysis), content validation (this type of validation can be done by inviting a panel of experts to review the items and different sections of the questionnaire), criterion (predictive) validation (the ability of the instrument to predict future behavior and attitudes; this type of validation can be done via correlation coefficients), and reliability tests (e.g. calculating Cronbach's alpha; for a discussion of these validation procedures, see Bachman, 1990; Drost, 2011; Newton and Shaw, 2014). This is because a measure cannot be considered valid without first being considered reliable (Pedhazur and Schmelkin, 1991). What follows is a description of the ways in which the various items and constructs that comprise the final version of the MULTITEACH questionnaire were developed, the validation procedures that were implemented, the results from the reliability tests, and a brief discussion of the findings as these concern language teacher multilingualism.

### 3.1 Developing the items: Phase 1

The theoretical basis for developing the questionnaire was found in works by Aronin (2017), Jessner (2008, 2018), and Cook (2008), which helped to identify the cognitive advantages and the language-specific and non-language-specific skills that multilingual language teachers can acquire as a result of their knowledge of multiple languages. In order to supplement these theoretical works, a large-scale empirical review of published studies on MLTs was subsequently conducted to aggregate and analyze the findings regarding their beliefs, identity, experiences, and use of multilingual practices with their students. The review (see Calafato, 2019), which assessed 84 empirical studies and focused on non-native speaker MLTs of various languages, helped to develop items for the MULTITEACH questionnaire that explored the diverse facets of language teacher multilingualism and which were supported by theoretical frameworks and empirical evidence. In addition, as recommended by Dörnyei (2003), the target group (language teachers in this case) was also invited to contribute to the questionnaire design process by sharing their ideas and offering feedback on the items generated via the empirical review.

This was accomplished via informal interviews and discussions with 24 MLTs who were contacted using convenience sampling (via social media, teacher forums, and acquaintances). The interviewees were from a variety of language backgrounds and were teaching in different contexts internationally. They were asked about their experiences learning multiple languages, their strategies for maintaining their proficiency in these languages, their beliefs about their affordances as MLTs, and their approaches to language teaching. The interviews and discussions were conducted in person or over Skype, depending on what was convenient for the teachers. Out of the 24 MLTs, 10 teachers discussed their experiences teaching multiple languages. The differences in their language backgrounds, gender, age, and teaching contexts helped to achieve variation regarding how they defined their multilingualism, language learning experiences, and practices in the classroom. The theoretical works, the empirical review, and the informal discussions with the 24 MLTs led to the compilation of an exploratory list of 90 statements that were converted into items for the MULTITEACH questionnaire, which would be administered online.

Of the 90 statements, 75 explored the participants' beliefs regarding the benefits of being/becoming multilingualism for their students and themselves, how strongly they believed multilingualism was promoted by parents and the state, their self-reported teaching ability per language taught, the competencies they believed a language teacher should possess, and how frequently they engaged in multilingual practices with their students. These statements were transformed into 6-point Likert items and each item was coded to assess the participants' agreement with a specific statement. The remaining 15 items consisted of a mix of Likert items and open-ended questions that covered the participants' language backgrounds, biographical data (gender, age, and teaching experience), language learning experiences, use of languages outside of work, the languages they taught, and their attitudes towards native-speakerism. These 15 items provided additional context to the questionnaire's exploration of language teacher multilingualism and comprised the main independent variables to be used during data analysis. Care was also taken to make sure that the questionnaire would take no more than 30 minutes to complete (see Dörnyei and Taguchi, 2009).

### 3.2 Seminar and consultations: Phase 2

Before piloting the questionnaire, it was translated using a multiple-forward translation procedure to make sure that the meaning and conceptual characterization of each questionnaire item was preserved (Sumathipala and Murray, 2000). The translations were done from English into Russian and Norwegian since the project would take place in Norway and Russia. It was decided early on that the participants should have the option to answer the questionnaire in their national languages in addition to English. This would ensure that they could answer the questionnaire in whatever language they felt comfortable, switching back and forth between English, Russian, and Norwegian in real time for any and all items and because completing the questionnaire in this way might end up requiring less time on their part. The translation process consisted of several phases. Firstly, two groups consisting of language teachers and university faculty members involved in foreign language didactics in Russia and Norway independently translated the questionnaire into Russian and Norwegian. Each group was composed of approximately four individuals. This phase resulted in eight translated versions (Russian and Norwegian) of the questionnaire, which were then compared with each other to identify differences in translation.

The groups were also asked for their feedback regarding the questionnaire's coherence, clarity, conceptual ambiguity, relevance to language teachers in Norway and Russia, and cohesion since there were teachers among the two groups who could provide valuable insights in this regard (Velayutham et al., 2011). With respect to the Russian questionnaire, inter-translator reliability was very high, with there being only four items where a slight difference in word choice was present among two of the translators. As for the Norwegian questionnaire, variances were slightly more pronounced, with differences at the phrasal level. These differences were collated together, after which the items were discussed with the Norwegian translators via email correspondence or face-to-face meetings in order to reach a consensus regarding the appropriate translated version. These consultations led to a final round of alterations to the Norwegian version. During this time, a seminar was also held with a panel of researchers studying multilingualism in educational settings in Norway to discuss their impressions of the questionnaire items. Their feedback was generally positive, although one of the researchers suggested a slight rewording of a pair of items in English.

Moreover, in discussions with the translators and researchers, it was decided that none of the items in the questionnaire would explicitly mention the words *multilingualism*, *bilingualism*, *multilingual*, or any other derivations thereof. Instead, the items would refer to competence in, and the learning of, multiple languages, implicitly drawing on the definition of multilingualism suggested by Clyne (2017). This decision was taken because the words *multilingualism* and *multilingual*, especially when translated into languages other than English, can take on a variety of additional meanings and social connotations. For example, some might define *multilingualism* as the speaking of multiple first languages (L1s) due to being born into a multicultural family (i.e. biographic multilingualism) as opposed to acquiring languages (i.e. acquired multilingualism) later in life (Kaçar and Bayyurt, 2018). *Flerspråklig*, which is the Norwegian equivalent of the term *multilingual,* can denote specifically immigrants or foreigners and not necessarily multilingual Norwegians. In this respect, feedback from the translators and researchers, who were proficient in English and Norwegian or Russian (and often one or two additional FLs), proved very helpful.

### 3.3 Piloting and teacher feedback: Phase 3

For the pilot study, an information sheet in Russian and Norwegian was sent out with an email to the administrative departments of upper-secondary schools in Bergen and Moscow, explaining the scope of the study and inviting language teachers to participate. The email contained a link to the MULTITEACH questionnaire and school officials were asked to forward the email to their language teaching staff so that they might participate in the project. Participation was voluntary and teachers were guaranteed anonymity and confidentiality. They were also informed that the purpose of the questionnaire was to understand teachers' approaches to the learning and teaching of multiple languages. The questionnaire, which was developed using SurveyXact, took about 20 minutes to complete and was left open for three weeks. At the end of this period, 57 language teachers (6 males; 36 females; 15 chose not to disclose their gender) had sent in completed questionnaires. Of these teachers, 27 were from Norway, and 30 were from Russia.

The participants reported teaching English, French, German, and Spanish as an FL. A small contingent from Norway reported teaching Norwegian as an L1 alongside one of the four abovementioned FLs. Some of the participants indicated that they were teaching multiple languages simultaneously. Since it was an online questionnaire, the responses were obtained instantaneously and were subsequently entered into a dataset that was analyzed using SPSS 25 and JASP. Several participants commented on the questionnaire's design and content in the space provided for comments at the end of the questionnaire, as well as via email and Skype. Their comments led to two main observations: 1) the questionnaire was interesting and relevant; 2) it was also very long and it would be better to remove some items, notably those on language teacher competencies. The participants felt that these items were repetitive, vague, and not specific to language teaching (i.e. they could be applied to all teachers). Their feedback was noted down in order to complement the subsequent statistical validation of the questionnaire.

## 4 Results

### 4.1 Principal Component Analysis and revising the questionnaire: Phase 4

**Figure 1:**
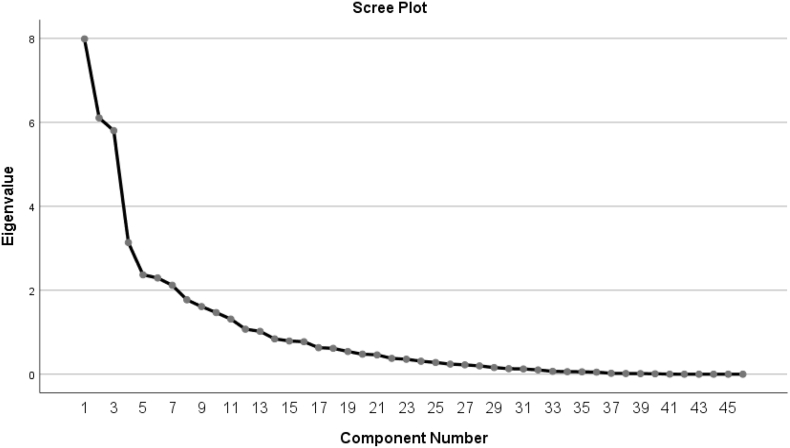
Scree plot for eigenvalues of components following PCA.

Principal Component Analysis (PCA) was performed in order to reduce the size of the questionnaire. Several items did not load saliently during the PCA and were, therefore, in combination with teacher feedback on the pilot, removed from the questionnaire. This resulted in a reduction of the Likert items on teacher multilingualism from 75 to 46. Figure 1 displays the scree plot for the eigenvalues of the principal components computed during the PCA. Component selection was carried out using the approach suggested by Cattell (1966), who recommended a cut-off where there is a noticeable break in the slope of the scree plot. In Figure 1, one can see this break occurring after the seventh component. The logic of this approach is that the break in the slope separates the major components from the minor ones (Cattell, 1966); parallel analysis can be used if the plot does not show a discernable break (see Karami, 2015), although this was not the case here. Each of the first seven components reported an eigenvalue of over 2, with the model explaining 64.81% of the variance.

Table 1 displays the component loadings for the 46 Likert items (a cut-off of .30 is used when displaying coefficient values).

**Table 1:** PCA component loadings for the 46 items on teacher multilingualism.

Items	Components						
	1	2	3	4	5	6	7
1. Learning multiple languages significantly improves one's intercultural competence.					.671		
2. It is possible to learn to speak, read and write in several foreign languages fluently.					.532		
3. Learning multiple languages improves one's cognitive skills.					.600		
4. Learning multiple languages can improve performance in Science, Math and Technology subjects.					.543		
5. Students who speak several languages can serve as linguistic role models for other learners.					.521		
6. Knowing multiple languages makes it easier to learn additional languages.					.448		
7. Learning additional languages improves knowledge of previously learned languages.					.577		
8. Learning multiple foreign languages simultaneously can hinder the language learning process.					-.563		.650
9. The presence of many foreign languages in a country can reduce the importance of national languages and associated cultures.							.348
10. It is better to learn one language at a time.					-.416		.723
11. Using languages other than the target language in lessons can cause confusion in students.					-.544		.608
12. One learns more effectively if only the target language is used during lessons.							.419
13. Parents promote their children's learning of multiple languages where I live.				.420			
14. The government promotes the learning of multiple languages where I live by providing sufficient time for language instruction in schools.				.712			
15. The government promotes the learning of multiple languages where I live by organizing campaigns that promote language learning.				.651			
16. The government promotes the learning of multiple languages where I live by investing money in language teacher education.				.887			
17. The government promotes the learning of multiple languages where I live by investing money in language materials				.858			
18. How easy do you find teaching Grammar?	.632						
19. How easy do you find teaching Vocabulary?	.767						
20. How easy do you find teaching Listening skills?	.811						
21. How easy do you find teaching Reading skills?	.704						
22. How easy do you find teaching Writing skills?	.755						
23. How easy do you find teaching Speaking skills?	.790						
24. How easy do you find teaching Cultural knowledge?	.635						
25. How easy do you find teaching Pronunciation?	.722						
26. How easy do you find teaching Language use in context (Pragmatics)?	.780						
27. The more languages teachers know, the better they can explain language structure.		.681					
28. The more languages teachers know, the better they can identify the language-related challenges learners face.		.712					
29. The more languages teachers know, the better they can use more appropriate teaching methods/approaches.		.858					
30. The more languages teachers know, the better they can increase their repertoire of activities.		.811					
31. The more languages teachers know, the better they can develop learners' intercultural competence.		.730					
32. The more languages teachers know, the better they can inspire students to learn languages.		.705					
33. I focus on explaining the structure of the language.			.715				
34. I focus on practicing communication and learning language structure more implicitly.			-.555				
35. I encourage students to translate from the target language during pair/group work.			.444				
36. I try to incorporate the other languages my students know or are learning into lessons.			.552				
37. I try to learn the other languages my students know and use these in my lessons.			.465				
38. I encourage students to use the other languages they know or are learning during lessons.			.610			.308	
39. I like to point out similarities and differences in the target language and the other languages my students and I know or are learning.			.747				
40. I give my students advice on how to understand certain concepts in the target language by relating them to the languages my students know or are learning.			.749				
41. I combine reading/listening activities in other languages that students know with speaking/writing activities in the target language.			.704				
42. I combine speaking/writing activities in other languages that students know with reading/listening activities in the target language.			.705				
43. I provide spaces where students and teachers can post content in different languages.						.600	
44. I display students' foreign language works in classrooms or elsewhere.						.605	
45. My students each have a language diary where they write their thoughts regarding the languages they are learning or are interested in.						.660	
46. I encourage my students to write texts using a combination of all the languages they already know or are learning.						.743	

The seven components thus identified were interpreted as follows: MLTB (items 1–7) represents the participants' beliefs about the benefits of being/becoming multilingual, MONO (items 8–12) represents their preference for a monolingual approach to language education, MLTSOC (items 13–17) comprises their beliefs regarding how strongly multilingualism is promoted by parents and the state, PROF (items 18–26) represents their self-reported teaching ability, MLTI (items 27–32) comprises their beliefs regarding MLT affordances, MTLS (items 33–42) encompasses their use of multilingual practices in the classroom, and GMLTP (items 42–46) concerns their more general attempts to promote an awareness of multilingualism among their students.

A reliability assessment, conducted using Cronbach's Alpha (α), McDonald's omega (ω), and Guttman's Lambda 4 (λ_4_), was subsequently undertaken following the item reduction initiated during the PCA. McDonald's ω and Guttman's λ_4_ are reported alongside α because, as has been pointed out by Sijtsma (2009), there is a tendency among researchers to use α as the sole indicator of reliability even though there are other, some might argue better, alternatives present (Benton, 2015; McNeish, 2018). Therefore, it was felt that the inclusion of more than one reliability estimate might reflect better practice in terms of providing a more complete picture of the components' reliability (Sijtsma, 2009). Pearson correlations (*r)* were also calculated to assess the strength and nature of the relationship between each of the identified components. Table 2 contains the descriptives, reliability test results (Cronbach's *α*, McDonald's ω, and Guttman's λ_4_), and Pearson correlations for the pilot questionnaire's seven components.

**Table 2:** Reliability statistics and Pearson correlations for the 7 components identified via PCA.

Component	Reliability						Pearson's Correlation (*r*)						
	n	M	SD	α	λ_4_	ω	1	2	3	4	5	6	7
1. MLTB	7	34.75	4.31	.836	.845	.851	-						
2. MONO	5	15.79	3.92	.616	.637	.627	-.195	-					
3. MLTSOC	5	14.71	4.15	.817	.706	.832	.187	.005	-				
4. PROF	9	36.74	8.79	.933	.898	.934	.175	-.310∗	.017	-			
5. MLTI	6	30.11	4.56	.903	.838	.909	.496∗∗	-.279	-.027	.044	-		
6. MLTS	10	30.42	9.34	.847	.725	.855	.237	.070	.295	.163	.631	-	
7. GMLTP	4	6.21	2.76	.695	.787	.685	.148	.143	.297	-.026	.010	.567∗∗	-

∗*p* < .05; ∗∗*p* < .01.

The Cronbach's alpha coefficients in Table 2 indicate that each of the components has a suitable level of reliability, based on the criteria put forward by Cohen et al. (2007), except for MONO, which has an α of .616. MONO's Guttman's split coefficient (λ_4_), however, indicates acceptable reliability (.637). In fact, the λ_4_ coefficient for each of the seven components shows an acceptable level of reliability (from .637 to .898), as does McDonald's omega. As for Pearson's correlation coefficient, the data from Table 2 indicate that the components are generally weakly correlated and that these correlations are not statistically significant in most instances, which suggests that the components are relatively independent and support the internal construct validity of the MULTITEACH questionnaire. Only in the case of MLTI and MLTB (*r* = .491), MONO and PROF (*r* = -.310), and MLTS and GMLTP (*r* = .567) are the correlations statistically significant and of somewhat medium strength.

### 4.2 Confirming the PCA: Phase 5

The MULTITEACH questionnaire was subsequently sent to upper-secondary schools in several counties in Norway and Russia, with language teachers invited to participate. A total of 460 language teachers of English, French, German, Spanish, Italian, and Chinese (231 from Russia and 229 from Norway) successfully completed the questionnaire, following which factor analysis (FA), using the Maximum Likelihood extraction and Direct Oblimin rotation methods, was performed. The Direct Oblimin method was selected because, being an oblique rotation method, it generally produces more accurate results than do orthogonal rotation methods when the underlying factors are correlated (Brown, 2006). Moreover, should no correlations exist between the underlying factors, oblique rotation methods produce similar results to those obtained through the use of orthogonal rotation methods. As a result, some researchers generally recommend using oblique rotation methods over orthogonal methods when conducting FA (e.g. Conway and Huffcutt, 2003). The FA factor loadings are listed in Table 3.

**Table 3:** FA factor loadings for the 46 items on teacher multilingualism.

Items	Factors						
	1	2	3	4	5	6	7
1. Learning multiple languages significantly improves one's intercultural competence.				.557			
2. It is possible to learn to speak, read and write in several foreign languages fluently.				.709		-.311	
3. Learning multiple languages improves one's cognitive skills.				.841			
4. Learning multiple languages can improve performance in Science, Math and Technology subjects.				.457			
5. Students who speak several languages can serve as linguistic role models for other learners.				.556			
6. Knowing multiple languages makes it easier to learn additional languages.				.566			
7. Learning additional languages improves knowledge of previously learned languages.				.527			
8. Learning multiple foreign languages simultaneously can hinder the language learning process.				-.524		.559	
9. The presence of many foreign languages in a country can reduce the importance of national languages and associated cultures.						.447	
10. It is better to learn one language at a time.						.628	
11. Using languages other than the target language in lessons can cause confusion in students.				-.339		.456	
12. One learns more effectively if only the target language is used during lessons.				-.496		.411	
13. Parents promote their children's learning of multiple languages where I live.					.536		
14. The government promotes the learning of multiple languages where I live by providing sufficient time for language instruction in schools.					.755		
15. The government promotes the learning of multiple languages where I live by organizing campaigns that promote language learning.					.695		
16. The government promotes the learning of multiple languages where I live by investing money in language teacher education.					.889		
17. The government promotes the learning of multiple languages where I live by investing money in language materials.					.860		
18. How easy do you find teaching Grammar?	.626						
19. How easy do you find teaching Vocabulary?	.759						
20. How easy do you find teaching Listening skills?	.816						
21. How easy do you find teaching Reading skills?	.728						
22. How easy do you find teaching Writing skills?	.750						
23. How easy do you find teaching Speaking skills?	.782						
24. How easy do you find teaching Cultural knowledge?	.631						
25. How easy do you find teaching Pronunciation?	.728						
26. How easy do you find teaching Language use in context (Pragmatics)?	.774						
27. The more languages teachers know, the better they can explain language structure.			.656				
28. The more languages teachers know, the better they can identify the language-related challenges learners face.			.717				
29. The more languages teachers know, the better they can use more appropriate teaching methods/approaches.			.850				
30. The more languages teachers know, the better they can increase their repertoire of activities.			.839				
31. The more languages teachers know, the better they can develop learners' intercultural competence.			.680				
32. The more languages teachers know, the better they can inspire students to learn languages.			.670				
33. I focus on explaining the structure of the language.		.654					
34. I focus on practicing communication and learning language structure more implicitly.		-.460					
35. I encourage students to translate from the target language during pair/group work.		.422					
36. I try to incorporate the other languages my students know or are learning into lessons.		.698					
37. I try to learn the other languages my students know and use these in my lessons.		.583					
38. I encourage students to use the other languages they know or are learning during lessons.		.673					
39. I like to point out similarities and differences in the target language and the other languages my students and I know or are learning.		.829					
40. I give my students advice on how to understand certain concepts in the target language by relating them to the languages my students know or are learning.		.789					
41. I combine reading/listening activities in other languages that students know with speaking/writing activities in the target language.		.751					.334
42. I combine speaking/writing activities in other languages that students know with reading/listening activities in the target language.		.744					.322
43. I provide spaces where students and teachers can post content in different languages.						-.337	.506
44. I display students' foreign language works in classrooms or elsewhere.						-.441	.353
45. My students each have a language diary where they write their thoughts regarding the languages they are learning or are interested in.							.409
46. I encourage my students to write texts using a combination of all the languages they already know or are learning.						-.358	.534

The results of the FA revealed that the model explained 61.89% of the variance (all the items loaded onto one or more of the seven factors; the eigenvalue for each factor was over 1). Kaiser-Meyer-Olkin (KMO) (.821) and Bartlett's test of sphericity [χ^2^ (1035) = 9105.31, p < .01] indicated that the sample was adequate for conducting factor analysis. RMSEA [.051; 90% CI = (.044, .052)], GFI (.975), and SRMR (.063) scores similarly pointed to a satisfactory fit (Schreiber et al., 2006).

Table 4 lists the descriptives, reliability test results, and Pearson correlations for the final questionnaire's seven constructs.

**Table 4:** Reliability statistics and Pearson correlations for the final questionnaire's 7 constructs.

Construct	Reliability						Pearson's Correlation (*r*)						
	n	M	SD	α	λ_4_	ω	1	2	3	4	5	6	7
1. MLTB	7	35.73	4.05	.758	.702	.752	-						
2. MONO	5	15.71	3.69	.652	.616	.673	-.265∗∗	-					
3. MLTSOC	5	15.75	4.60	.781	.739	.807	.105	.076	-				
4. PROF	9	39.86	58.84	.897	.863	.898	.253∗∗	-.106∗	.106∗	-			
5. MLTI	6	30.23	4.48	.868	.838	.864	.483∗∗	-.104∗	.020	.239∗∗	-		
6. MLTS	10	30.70	8.88	.799	.711	.816	.130∗∗	-.167∗∗	-.047	.139∗∗	.268∗∗	-	
7. GMLTP	4	6.78	3.28	.658	.656	.674	.051	-.127∗	.127	..028	.130∗	.439∗∗	-

∗*p* < .05; ∗∗*p* < .01.

As the reliability coefficients in Table 4 indicate, each of the constructs, similar to the reliability scores obtained for the pilot questionnaire's 7 components, has a suitable level of reliability. As for Pearson's correlation coefficient, the data from Table 4 reveal that statistically significant, weak, negative correlations exist between MLTB, MLTI, GMLTP, PROF, and MLTS, on the one hand, and MONO, on the other. Statistically significant, weak, positive correlations also exist between MLTI, MLTS, and GMLTP. Identical to the findings from the pilot questionnaire, statistically significant, medium-strength, positive correlations were found between MLTB and MLTI, as well as between MLTS and GMLTP.

## 5 Discussion

This study described the procedures undertaken to help ensure that the MULTITEACH questionnaire on teacher multilingualism was validated using a number of approaches and that it was sufficiently reliable.

With respect to the questionnaire's construct and criterion validity, results from the PCA, reliability and correlation test procedures, and FA indicated that the constructs were relatively independent and had high internal consistency, with items generally having high factor loadings. As for content validation, the critical review of empirical studies (Calafato, 2019), the consultations with the translator groups and teachers during the entire process of formulating and piloting the questionnaire, the seminar with the panel of researchers, and the interviews with MLTs helped to ensure that the conditions for content validity were met. In terms of the questionnaire's predictive ability regarding language teacher multilingualism, a number of statistically significant correlations were found between the various MULTITEACH constructs. Firstly, the participants' beliefs about the affordances of MLTs (MLTI) statistically significantly and positively correlated with their beliefs regarding the benefits of being/becoming multilingual (MLTB). Moreover, MLTI and MLTB statistically significantly and negatively correlated with the participants' preference for a monolingual approach to language teaching (MONO) (see Tables 2 and 4). The findings support those from other studies where it was found that MLTs did not view their and their students' multilingualism as a resource if they subscribed to a monolingual approach to teaching languages (see Zheng, 2017).

MLTB and MLTI also statistically significantly and positively, albeit weakly, correlated with the extent to which the participants reported engaging in multilingual practices with their students (MLTS) (see Table 4). Previous studies have shown that positive beliefs about multilingualism do not always lead to teachers adopting a multilingual pedagogy (e.g. Haukås, 2016). Here, the correlations were quite weak, perhaps because the teacher education programs the participants completed during their preservice years did not include an explicit component for developing their ability to engage in multilingual practices with their students (see Otwinowska, 2017). In the future, should teacher education programs take steps to incorporate an explicit component on multilingual practices (see Raud and Orehhova, 2020), it is likely that there will be a stronger, more positive correlation between MLTB, MLTI, MLTS, and GMLTP. A stronger correlation between these constructs might also be achieved via deeper engagement with inservice teachers regarding the benefits of being/becoming multilingual (for both students and teachers). Such an engagement could be realized by organizing workshops on implementing a multilingual pedagogy and by conducting action research that promotes the implementation of multilingual practices among inservice teachers (see Calafato, 2019). Absent such initiatives, some language teachers might continue to be influenced by a monolingual approach to teaching languages that may negatively affect their ability and desire to implement multilingual practices in the classroom. The findings indicate as much, with MONO statistically significantly and negatively correlating with both MLTS and GMLTP.

Finally, the participants' self-assessment of their teaching ability (PROF) was found to statistically significantly and positively correlate with MLTB, MLTI, and MLTS. It also statistically significantly and negatively correlated with MONO, suggesting that the higher the participants rated their teaching ability, the stronger was their implementation of multilingual practices during lessons. Those who were less confident in their teaching ability were more likely to prefer a monolingual approach to teaching languages. The correlations are weak in each case, although they support the findings from other studies that have indicated a strong link between teachers' self-reported proficiency and their plurilingual awareness (e.g. Otwinowska, 2014). Language teachers who reported possessing a more advanced level of teaching ability were likely more aware of the languages they taught and more confident when drawing on their knowledge of language to engage in multilingual practices with their students. Those participants who reported facing difficulties teaching certain aspects of the target language might have had limited knowledge of, and insights into, the languages they taught. As a result, they engaged in multilingual practices less frequently. As such, the findings indicate that, in addition to engaging with teachers regarding their beliefs about the benefits of being/becoming multilingual, there is a need to focus on further developing their teaching abilities since the participants' perception of these positively correlated with their reported implementation of multilingual practices during lessons.

## 6 Conclusion

The MULTITEACH questionnaire represents one of the very few attempts to create a measure that can help institutions and researchers involved in the study and promotion of multilingual initiatives and associated teacher education programs to evaluate language teacher multilingualism on a large scale. The measure, in its current state, not only has acceptable levels of reliability and cohesiveness but it has also been validated using a variety of approaches that, most importantly, included input from language teachers. In other words, MULTITEACH is an evaluative instrument for language teachersthat has been partly developed by language teachers. At the same time, as with all such instruments, it is important to conduct additional studies with the MULTITEACH questionnaire in other countries and in other teaching contexts, ideally in conjunction with interviews and participant observations, in order to better assess its reliability as a measure that can evaluate teacher multilingualism. It is possible, for example, that language teachers from countries like China and the UK will respond to MULTITEACH in a very different way than did the participants from Norway and Russia in this study. This makes MULTITEACH a useful tool for researchers when carrying out cross-cultural research into language teacher multilingualism.

It is also hoped that MULTITEACH will serve as a basis for evaluating teacher multilingualism in non-language subjects, something which can be achieved with slight modifications. In conclusion, the MULTITEACH questionnaire provides educational institutions, governments, and researchers with the possibility to conveniently and comprehensively evaluate language teacher multilingualism. The insights thus obtained can help them to design and implement teacher education programs that create greater synergy between teacher beliefs and practices, something which has not always been done systematically in many teacher education programs until now. The need for such an evaluative measure will likely only grow stronger as classrooms around the world become more and more super-diverse and countries place ever greater emphasis on developing multilingual citizens who can thrive in an increasingly globalized, multipolar world. In this regard, a vital first step is for educational institutions and countries to be able to evaluate language teacher multilingualism in a timely and comprehensive manner so that they obtain relevant and accurate information that allows them to then help teachers develop the knowledge and skills necessary to ensure that they and their students realize their multilingual potential.

## Declarations

### Author contribution statement

R. Calafato: Conceived and designed the experiments; Performed the experiments; Analyzed and interpreted the data; Contributed reagents, materials, analysis tools or data; Wrote the paper.

### Funding statement

This research did not receive any specific grant from funding agencies in the public, commercial, or not-for-profit sectors.

### Competing interest statement

The authors declare no conflict of interest.

### Additional information

No additional information is available for this paper.

## Appendix A Supplementary data

The MULTITEACH questionnaire The MULTITEACH questionnaire
