# Supplementary material for: Evaluating teacher multilingualism across contexts and multiple languages: validation and insights
Source: Heliyon. 2020 Aug 7;6(8):e04471. doi: 10.1016/j.heliyon.2020.e04471 (PMC7415832; doi:10.1016/j.heliyon.2020.e04471)
Supplement: The MULTITEACH questionnaire [file mmc1.docx]

**The MULTITEACH questionnaire**

| **Section 1. Language learning background**  This section contains questions about your language background. There are no right or wrong answers. We are interested in your thoughts and experiences. You can change the language at any time (look for the options at the bottom of each page). |
| --- |

1. What is your mother tongue? If you think you have more than one mother tongue, please list them below as well.
2. What languages do you speak in your free time? Please list them below.
3. What languages do you feel you can express yourself freely in? Please list them below.
4. Which languages, if any, did you study in school and university? Please list them in the appropriate boxes below.

| School |  |
| --- | --- |
| University |  |

1. Have you studied or are you studying any languages on your own? If yes, please list them in the corresponding spaces below and describe how you primarily studied them or are studying them (e.g. online, self-study manual, etc.).

| Previously learned languages |  |
| --- | --- |
| Currently learning |  |

| **Section 2. Language teaching background**  This section contains questions about your language teaching background. |
| --- |

1. How long have you been a language teacher?

❑ Less than a year

❑ 1 - 2 years

❑ 3 - 4 years

❑ 5 - 9 years

❑ 10 years or over

1. Select the language(s) you are currently teaching.

❑ Norwegian/Russian

❑ English

❑ French

❑ German

❑ Spanish

❑ Italian

❑ Chinese

1. Do you use (Lx) outside of school hours?

❑ Less than once a month

❑ Once a month

❑ Once every two weeks

❑ Once a week

❑ More than once a week but not daily

❑ Daily

1. Have you taught any other languages? If yes, please list them (and the context) below.

| **Section 3. Beliefs about language learning and teaching**  This section contains questions about your beliefs regarding language learning and teaching. |
| --- |

1. To what extent do you agree with the following statements?

|  | Strongly disagree | Disagree | Somewhat disagree | Somewhat agree | Agree | Strongly agree |
| --- | --- | --- | --- | --- | --- | --- |
| 10.1 Learning multiple languages significantly improves one's intercultural competence. | ❑ | ❑ | ❑ | ❑ | ❑ | ❑ |
| 10.2 It is possible to learn to speak, read and write in several foreign languages fluently. | ❑ | ❑ | ❑ | ❑ | ❑ | ❑ |
| 10.3 Learning multiple languages improves one's cognitive skills. | ❑ | ❑ | ❑ | ❑ | ❑ | ❑ |
| 10.4 Learning multiple foreign languages simultaneously can hinder the language learning process. | ❑ | ❑ | ❑ | ❑ | ❑ | ❑ |
| 10.5 Learning multiple languages can improve performance in Science, Math and Technology subjects. | ❑ | ❑ | ❑ | ❑ | ❑ | ❑ |
| 10.6 The presence of many foreign languages in a country can reduce the importance of national languages and associated cultures. | ❑ | ❑ | ❑ | ❑ | ❑ | ❑ |

1. Would you prefer a native speaker or non-native speaker teacher when learning a new language (all else being equal, e.g. qualifications, etc.)? Why?
2. Parents promote their children's learning of multiple languages where I live.

❑ Strongly disagree

❑ Disagree

❑ Somewhat disagree

❑ Somewhat agree

❑ Agree

❑ Strongly agree

1. The government promotes the learning of multiple languages where I live by…

|  | Strongly disagree | Disagree | Somewhat disagree | Somewhat agree | Agree | Strongly agree |
| --- | --- | --- | --- | --- | --- | --- |
| 13.1 …providing sufficient time for language instruction in schools | ❑ | ❑ | ❑ | ❑ | ❑ | ❑ |
| 13.2 …organizing campaigns that promote language learning | ❑ | ❑ | ❑ | ❑ | ❑ | ❑ |
| 13.3 …investing money in language teacher education | ❑ | ❑ | ❑ | ❑ | ❑ | ❑ |
| 13.4 …investing money in language materials | ❑ | ❑ | ❑ | ❑ | ❑ | ❑ |

1. How much do you agree with the following statements?

|  | Strongly disagree | Disagree | Somewhat disagree | Somewhat agree | Agree | Strongly agree |
| --- | --- | --- | --- | --- | --- | --- |
| 14.1 It is better to learn one language at a time. | ❑ | ❑ | ❑ | ❑ | ❑ | ❑ |
| 14.2 Students who speak several languages can serve as linguistic role models for other learners. | ❑ | ❑ | ❑ | ❑ | ❑ | ❑ |
| 14.3 Using languages other than the target language in lessons can cause confusion in students. | ❑ | ❑ | ❑ | ❑ | ❑ | ❑ |
| 14.4 Knowing multiple languages makes it easier to learn additional languages. | ❑ | ❑ | ❑ | ❑ | ❑ | ❑ |
| 14.5 One learns more effectively if only the target language is used during lessons. | ❑ | ❑ | ❑ | ❑ | ❑ | ❑ |
| 14.6 Learning additional languages improves knowledge of previously learned languages. | ❑ | ❑ | ❑ | ❑ | ❑ | ❑ |

1. How easy do you find teaching the following in (Lx)?

|  | Very difficult | Difficult | Somewhat difficult | Somewhat easy | Easy | Very easy |
| --- | --- | --- | --- | --- | --- | --- |
| 15.1 Grammar | ❑ | ❑ | ❑ | ❑ | ❑ | ❑ |
| 15.2 Vocabulary | ❑ | ❑ | ❑ | ❑ | ❑ | ❑ |
| 15.3 Listening skills | ❑ | ❑ | ❑ | ❑ | ❑ | ❑ |
| 15.4 Reading skills | ❑ | ❑ | ❑ | ❑ | ❑ | ❑ |
| 15.5 Writing skills | ❑ | ❑ | ❑ | ❑ | ❑ | ❑ |
| 15.6 Speaking skills | ❑ | ❑ | ❑ | ❑ | ❑ | ❑ |
| 15.7 Cultural knowledge | ❑ | ❑ | ❑ | ❑ | ❑ | ❑ |
| 15.8 Pronunciation | ❑ | ❑ | ❑ | ❑ | ❑ | ❑ |
| 15.9 Language use in context (Pragmatics) | ❑ | ❑ | ❑ | ❑ | ❑ | ❑ |

1. The more languages teachers know, the better they can...

|  | Strongly disagree | Disagree | Somewhat disagree | Somewhat agree | Agree | Strongly agree |
| --- | --- | --- | --- | --- | --- | --- |
| 16.1 …explain language structure | ❑ | ❑ | ❑ | ❑ | ❑ | ❑ |
| 16.2 …identify the language-related challenges learners face | ❑ | ❑ | ❑ | ❑ | ❑ | ❑ |
| 16.3 …use more appropriate teaching methods/approaches | ❑ | ❑ | ❑ | ❑ | ❑ | ❑ |
| 16.4 …increase their repertoire of activities | ❑ | ❑ | ❑ | ❑ | ❑ | ❑ |
| 16.5 …develop learners' intercultural competence | ❑ | ❑ | ❑ | ❑ | ❑ | ❑ |
| 16.6 …inspire students to learn languages | ❑ | ❑ | ❑ | ❑ | ❑ | ❑ |

1. I am aware of all the languages each of my students can make themselves understood in.

❑ I don't know this about any student

❑ I know this about some students (25% of them)

❑ I know this about quite a few students (50% of them)

❑ I know this about many students (75% of them)

❑ I know this about all my students

| **Section 4. Teaching methods and activities**  This section contains questions about teaching methods and activities. |
| --- |

1. How often do you do the following during a typical month when teaching (Lx)?

|  | Never | Once | Rarely | Sometimes | Often | Every lesson |
| --- | --- | --- | --- | --- | --- | --- |
| 18.1 I focus on explaining the structure of the language. | ❑ | ❑ | ❑ | ❑ | ❑ | ❑ |
| 18.2 I focus on practicing communication and teaching language structure more implicitly. | ❑ | ❑ | ❑ | ❑ | ❑ | ❑ |
| 18.3 I encourage students to translate from the target language during pair/group work. | ❑ | ❑ | ❑ | ❑ | ❑ | ❑ |
| 18.4 I try to incorporate the other languages my students know or are learning into lessons. | ❑ | ❑ | ❑ | ❑ | ❑ | ❑ |
| 18.5 I try to learn the other languages my students know and use these in my lessons. | ❑ | ❑ | ❑ | ❑ | ❑ | ❑ |
| 18.6 I encourage students to use the other languages they know or are learning during lessons. | ❑ | ❑ | ❑ | ❑ | ❑ | ❑ |
| 18.7 I like to point out similarities and differences in the target language and the other languages my students and I know or are learning. | ❑ | ❑ | ❑ | ❑ | ❑ | ❑ |
| 18.8 I give my students advice on how to understand certain concepts in the target language by relating them to the languages my students know or are learning. | ❑ | ❑ | ❑ | ❑ | ❑ | ❑ |
| 18.9 I combine reading/listening activities in other languages that students know with speaking/writing activities in the target language. | ❑ | ❑ | ❑ | ❑ | ❑ | ❑ |
| 18.10 I combine speaking/writing activities in other languages that students know with reading/listening activities in the target language. | ❑ | ❑ | ❑ | ❑ | ❑ | ❑ |

1. How often do you do the following during a typical month?

|  | Never | Once | Rarely | Sometimes | Often | Every lesson |
| --- | --- | --- | --- | --- | --- | --- |
| 19.1 I provide spaces where students and teachers can post content in different languages. | ❑ | ❑ | ❑ | ❑ | ❑ | ❑ |
| 19.2 I display students' foreign language works in classrooms or elsewhere. | ❑ | ❑ | ❑ | ❑ | ❑ | ❑ |
| 19.3 My students each have a language diary where they write their thoughts regarding the languages they are learning or are interested in. | ❑ | ❑ | ❑ | ❑ | ❑ | ❑ |
| 19.4 I encourage my students to write texts using a combination of all the languages they already know or are learning. | ❑ | ❑ | ❑ | ❑ | ❑ | ❑ |

1. Have you initiated any activities involving the use of two or more languages at your school? If yes, could you describe them briefly?
2. Has your school initiated any activities involving the use of two or more languages? If yes, could you describe them briefly?

| **Section 5. Biographical information**  This section questions about your age group and gender. |
| --- |

1. What is your gender? Choose an option.

❑ Male

❑ Female

❑ Prefer not to say

1. Choose the age group you belong to.

❑ 20-29

❑ 30-39

❑ 40-49

❑ 50-59

❑ 60-69
